# Exploring the Smallest Active Fragment of HsQSOX1b and Finding a Highly Efficient Oxidative Engine

**DOI:** 10.1371/journal.pone.0040935

**Published:** 2012-07-20

**Authors:** Wenyun Zheng, Wenyao Zhang, Wei Hu, Chao Zhang, Yi Yang

**Affiliations:** Synthetic Biology and Biotechnology Laboratory, State Key Laboratory of Bioreactor Engineering, School of Pharmacy, East China University of Science and Technology, Shanghai, China; Instituto de Biociencias - Universidade de São Paulo, Brazil

## Abstract

Human quiescin-sulfhydryl oxidase 1 isoform b (HsQSOX1b) is a highly efficient, multiple-domain enzyme that directly inserts disulfide bonds into client protein. However, previous studies have focused mainly on the catalytic activity of the whole protein rather than its domain structure. In this research, we dissected the structure and function of HsQSOX1b and explored its mechanism as a highly efficient sulfhydryl oxidase by analyzing the truncated variants. The results showed that the first HsQSOX1b thioredoxin domain was essential for thiol oxidase activity. The smallest active fragment (SAQ) was identified to consist of a helix-rich region (HRR) and an essential for respiration and viability/augmenter of liver regeneration (ERV/ALR) domain, which remained highly active to oxidize an artificial non-thiol substrate but not small molecular and protein thiols. Our study clearly demonstrated that SAQ is a highly efficient oxidative engine, which shows high efficiency in the *de novo* disulfide formation and oxygen reduction and that this more efficient oxidative engine is necessary for the highly efficient catalysis of QSOXs compared to Erv1 and Erv2. This study will help address the roles of different HsQSOX1b domains in *de novo* disulfide formation and encourage the engineering of more efficient QSOX variants for the *in vitro* folding of disulfide-containing proteins.

## Introduction

Quiescin-sulfhydryl oxidase are present in most eukaryotes [1], [2], [3] but not in yeast/fungi [4]. These oxidases possess similar structure and distribution and are predominantly found in the Golgi and secretory granules. A significant fraction of these oxidases is secreted from cells, and has been detected in the endoplasmic reticulum [5], [6], [7].

The relationship between the structure and function of QSOXs has been an important topic in exploring the disulfide-generating module and molecular mechanism on *in vivo* catalytic oxidation [8], [9]. However, previous studies have focused mainly on the catalytic activity of the whole protein rather than its domain structure [7], [10]. QSOXs belong to a group of enzymes called sulfhydryl oxidases, which includes the flavin adenine dinucleotide (FAD) prosthetic group, and oxidize small molecular thiol-containing substrate, i.g. dithiothreitol (DTT) and glutathione (GSH) [1], [11]. Among these enzymes, only QSOXs are capable of the facile and direct insertion of disulfide bonds into reduced client proteins, which is termed protein thiol oxidase activity. We previously reported that sulfhydryl oxidases, including QSOXs, are also capable of efficiently oxidizing non-thiol reductant substrates [12], such as tris [2-carboxyethyl] phosphine (TCEP), a specific reductant for disulfide. TCEP has been widely used for reduction of disulfide containing proteins and directly reduces the *de novo* formed disulfides residing in the active sites of these enzymes, thus regenerates free thiols, and makes the enzyme cycling and generating hydrogen peroxide from oxygen [12], [13]. Such activity was termed as TCEP oxidase activity. Moreover, Comparing to endoplasmic reticulum oxidoreductin 1 (Ero1) and Erv family of sulfhydryl oxidases, QSOXs have shown hundred-fold higher thiol oxidase and TCEP oxidase activities compared with the Ero1 and Erv families of sulfhydryl oxidases [10], [12].

**Figure 1:**
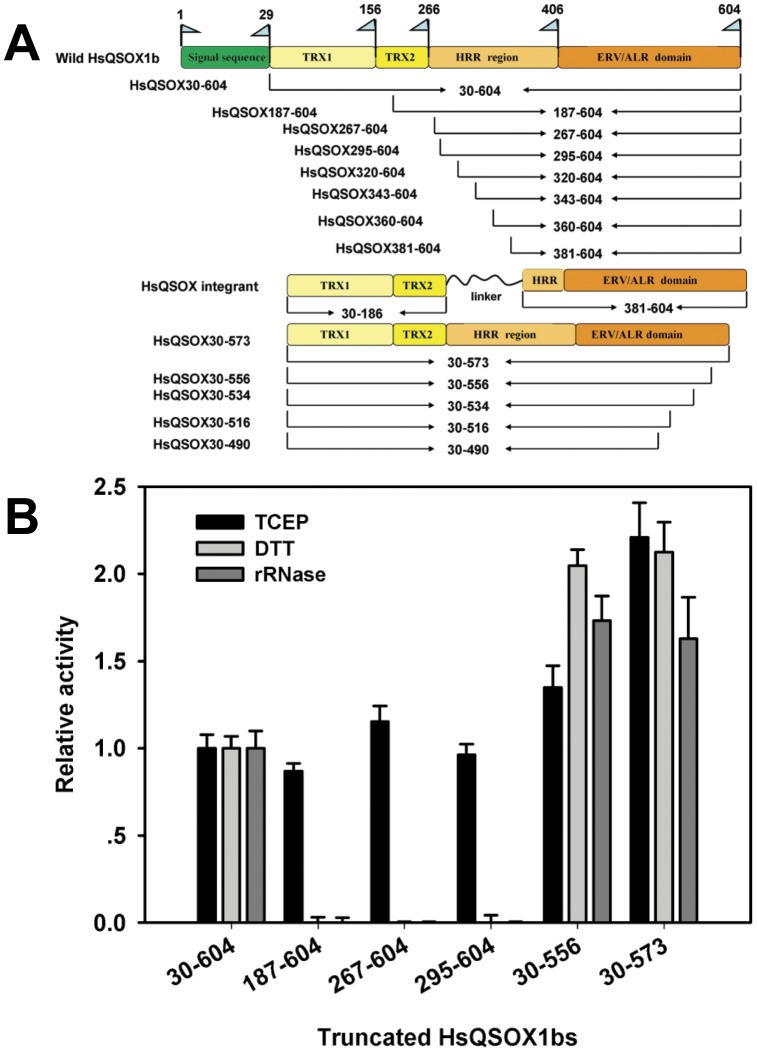
Schematic diagram of HsQSOX1b truncation and activity of the partial truncated variants. (A) A series of truncated enzymes were prepared according to the nine QHZs. Their secondary structures were verified through crystallization and prediction, and their names were formulated according to their amino acid boundaries. (B) The oxidase activities of HsQSOX1b truncated variants toward TCEP, DTT and rRNase, relative to those of the wildtype enzyme, respectively. The oxidase activity was based on determining the rate of the H_2_O_2_ generation [22]. The N-terminal truncated variants, namely, HsQSOX1b_187–604_, HsQSOX1b_267–604_, and HsQSOX1b_295–604_, retained their oxidase activity to TCEP but lost their thiol oxidase activity to reduced RNase and DTT. HsQSOX1b_30–573_ and HsQSOX1b_30–556_ had higher thiol oxidase activity than the full-length enzyme, and both the thiol and TCEP oxidase activities of HsQSOX1b_30–573_ were higher than those of HsQSOX1b_30–604_ and HsQSOX1b_30–556_.

Most QSOXs consist of four recognizable domains: two thioredoxin (Trx) domains, a helix-rich region (HRR), and an ERV/ALR domain. However, QSOXs from protozoan parasites lack the Trx2 domain (Fig. 1A) [14], [15]. *Thorpe* et al. [10] and our lab [12] have investigated the functions of three conserved CXXC motifs and the catalytic mechanism of HsQSOX1b, through site-directed mutation, which laid the foundation for a deeper understanding of the multiple-functions of its multiple domains. The results of the site-directed mutation of the C70–C73 motif indicated that the active site of the Trx1 domain did not participate in oxidizing the non-thiol substrates [12]. TCEP was directly oxidized by the critical C449–C452 motif located in the active site of QSOXs [12], suggesting that TCEP is a well substrate for evaluating the efficiency of de novo disulfide formation and oxygen reduction of QSOXs, i.e. the electron transfer from the C449–C452 motif to FAD and then to oxygen. However, the detailed functions that correspond to different domains remained unclear, and the mechanism of QSOX as a highly efficient sulfhydryl oxidase than the Erv1 and Ero1 family thiol oxidases was still unknown. A HsQSOX1b_286–546_ fragment was recently crystallized, and its structure resolved [16], showing that the HRR and ERV/ALR domain exist as an intrachain pseudo-dimer similar to the Erv2p homodimer. However, no studies have reported whether such a fragment retained the catalytic activity and whether its structure reflected the mechanism of a highly efficient enzyme. Thus, the corresponding function of the different HsQSOX1b domains in oxidase activity, particularly HRR, is still unknown.

The physiological function of QSOXs is to insert disulfide bonds to reduced polypeptide chains. This involves multiple steps, including disulfide generation in the core, disulfide transfer to C70–C73 motif, and disulfide insertion to a reduce protein [10], [15]. Previous study has demonstrated that the electron transfer pathway from the Trx1 domain to the Erv domain is critical and that ERV and Trx domains cooperate for “high efficiency” of QSOXs to oxidize reduced peptide or DTT, as shown by Heckler et al that mutation of either the C70–C73 motif or C449–C452 motif greatly decreased the activity toward reduced peptide or DTT [10], [12]. However, whether other steps, in particular disulfide generation in the core, contribute to high efficiency of QSOXs is still not clear.

To dissect the structure and function of this multi-domain, multi-function protein, we prepared a series of truncated variants of HsQSOX1b and examined their activities toward small molecular thiols, protein thiols, and TCEP. We found the smallest active fragment (SAQ) of HsQSOX1b, which consisted of the HRR and ERV/ALR domain. This fragment has shown very high TCEP oxidase activity, compared with the Erv family enzyme. To further evaluate the contribution of the *de novo* disulfide formation and oxygen reduction in the high efficiency of QSOXs, the oxidation of TCEP catalyzed by SAQ was analyzed. The high efficiency of SAQ in catalysis indicate that this much more efficient oxidative engine contribute to the highly efficient catalysis of QSOXs comparing to Erv1 and Erv2. Moreover, these studies provide a new insight into this enzyme for different types of substrates and encourage the development of sulfhydyl oxidase derivatives for assisting *in vivo* and *in vitro* protein folding.

## Results and Discussion

### The QSOX Fragment Lacking Trx Domains Lost Thiol Oxidase Activity but Retained TCEP Oxidase Activity

Three N-terminal truncated variants were designed based on the predicted nine quiescin homology zones (QHZs) [1]. HsQSOX1b_187–604_, without the Trx1 domain, was obtained by cutting QHZ0 and QHZ1. HsQSOX1b_381–604_, without the Trx1, Trx2 domains and HRR, was obtained by cutting the QHZ0–4. The two Trx domains, which were directly linked to the ERV/ALR domain by GAGAGA, resulted in the HsQSOX1b integrant without HRR (Fig. 1A). HsQSOX1b_267–604_, HsQSOX1b_320–604_, HsQSOX1b_343–604_ and HsQSOX1b_360–604_ were designed as predicted in the previous paper [15].

These above-mentioned truncated variants were analyzed to evaluate the function of the Trx domains on the catalytic activity of HsQSOX1b (Text S3). The results showed that the variants without Trx1 domain lost the capacity to oxidize DTT or reduced RNase (rRNase) completely (Fig. 1B and Table S2). On the other hand, a previous study showed that the activity of the C70A–C73A mutant variants retained the low yet substantial thiol oxidase activity to DTT and rRNase [10], [12]. These results indicated that the Trx1 domain is essential for the oxidation of either small molecular or proteineous thiol substrates, whereas the C70A–C73A motif significantly enhanced catalytic efficiency. Therefore, the thiol-oxidation activity of HsQSOX1b relies on the interaction of the Trx domains and ERV/ALR domain. Meanwhile, the thiol and protein thiol oxidase activities of HsQSOX1b without Trx domains could not be detected. We also found that the variants without two Trx domains showed the similar TCEP oxidase activity to the full-length enzyme, indicating that those two domains did not affect the folding of the other part of HsQSOX1b (Fig. 1B). Thus, no synergy exist between the Trx and ERV/ALR domain with respect to TCEP, neither the shuttling of the electron between the C70–C73 motif and the active site of ERV/ALR domain nor the fold between the Trx domains and the ERV/ALR domain. Furthermore, the variants without both Trx domains showed a similar TCEP oxidase activity to that of the Trx1-domain-lacking enzyme (Fig. 1B), suggesting that the Trx2 domain, without any redox-active disulfide, may not participate in enzyme catalysis. These agree with the facts that single-Trx QSOXs naturally exist [14].

HsQSOX1b was further truncated by removing the complete or partial HRR sequence to explore the function of HRR. We found that the yields of HsQSOX1b_320–604_, HsQSOX1b_343–604_, and HsQSOX1b_381–604_ was very low, which were different from the high yields of HsQSOX1b_187–604_, HsQSOX1b_267–604_, and the wildtype enzyme (Fig. S1). In addition, UV/visible spectra analysis revealed that HsQSOX1b_320–604_ lost its ability to bind FAD (Fig. S2). The results of the activity assay showed that the ERV/ALR domain of HsQSOX1b, which does not have a complete HRR (HsQSOX1b_381–604_), or its variants with a large part of the HRR removed (HsQSOX1b_320–604_, HsQSOX1b_343–604_ and HsQSOX1b_360–604_), have no oxidase activity toward DTT or TCEP (Fig. 1B and Table S2). Thus, these variants did not fold well, could not bind FAD, and thus lost any oxidase activity, and were easily degraded, suggesting that the folding of the HsQSOX1b ERV/ALR domain relied on the HRR domain. The structure of the HRR domain was very similar to that of Erv1p but without the FAD cofactor and catalytic activity [16]. Alon et al. [16] suggested that the HRR domain may play a critical role in maintaining the conformation of the active center through the formation of a pseudo-dimer with the ERV/ALR domain of HsQSOX1b.

### C-terminal Tail of HsQSOX1b Regulated the Catalytic Activity

To further dissect structure and function of HsQSOX1b, we designed the C-terminal truncated proteins according to the secondary structure verified via prediction [15] (Fig. 1A). In addition, the activity of the C-terminal truncated variants was also analyzed (Fig. 1B and Table S2). The deletion of 30 to 50 amino acid residues resulted in a roughly two-fold higher oxidase activity toward small molecular thiols, proteineous thiols, or TCEP (Fig. 1B). The result also showed that the TCEP oxidase activity of HsQSOX1b_30–556_ was lower than that of HsQSOX1b_30–573_, whereas the thiol and protein thiol oxidase activities of these two variants were very similar (Fig. 1B). As our previous work and the present study have shown, TCEP was oxidized in the active site of the QSOX ERV/ALR domain, while the thiol substrate was oxidized by the enzyme via C70–C73 motif in Trx domain [10], [12]. The C-terminal truncation from 573 to 556 may have different effects on the binding of these substrates and affects the enzyme activity. These results suggest that the C-terminal tail of QSOXs regulated the catalytic activity. The truncated variants without helix α5 (HsQSOX1b_30–516_) or part of it (HsQSOX1b_30–534_), which was located immediately downstream of the C509–C512 motif, showed the low yields and did not bind FAD, and thus showed the minimal or no activity (Fig. S1 and Table S2). Thus, the α5 helix is essential for the integrity of the HsQSOX1b structure and enzyme activity.

To explore the function of the C509–C512 motif, we constructed the HsQSOX1b_30–556_ through C509S/C512S mutation. The mutation of the C509–C512 motif did not affect the oxidation of TCEP but decreased the thiol and protein thiol activities (Fig. S3). Therefore the C509–C512 motif did not participate in the oxidation of TCEP, which was consistent with the result that TCEP was directly oxidized by the active site of the ERV/ALR domain. This result also suggest that the C509–C512 motif participate in the oxidation of thiol and proteineous thiol substrates for QSOXs, but not critical, which was consistent with the previous study [10]. However, how the C509–C512 motif assists the oxidation of thiol and proteineous thiol substrates and how the C-terminal tail of QSOX affects the oxidase activity were still unknown.

### SAQ of HsQSOX1b

**Figure 2:**
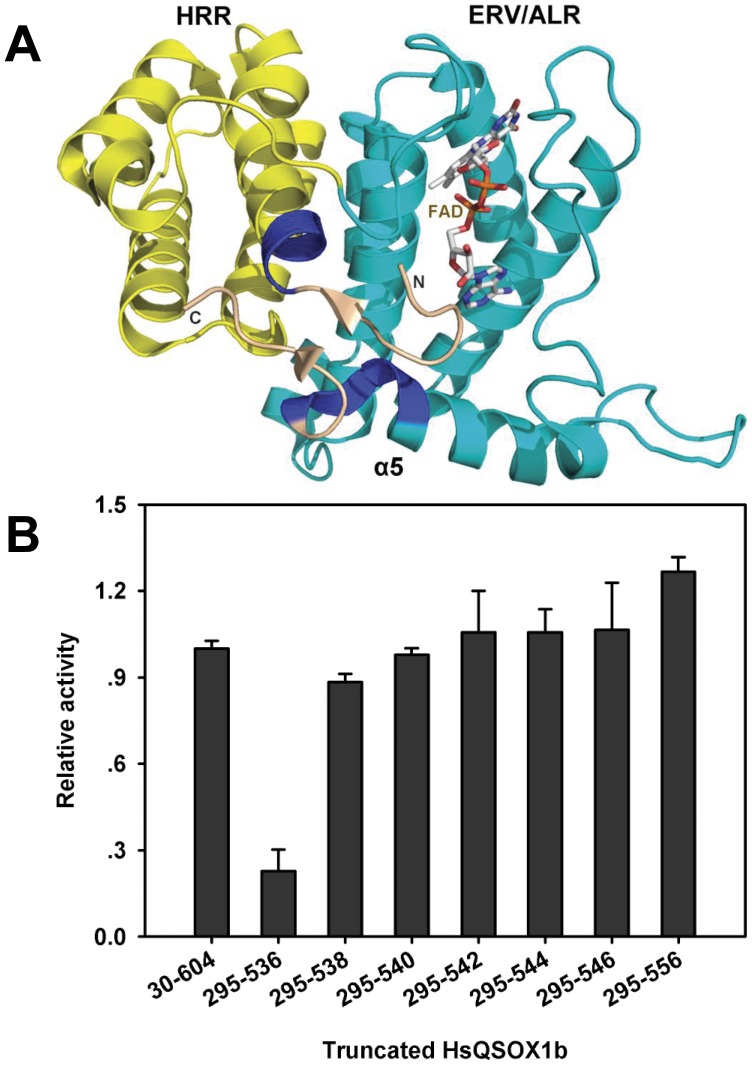
Crucial regions resolving HsQSOX activity. (A) Crystal structure of HsQSOX1b protein. The yellow helix represents the HRR (excluding the M301-L304 region), and the cyan helix represents the ERV/ALR domain (excluding the H534-S538 region). The blue region represents M301-L304 and H534-S538. Deletion both regions acutely decrease the activity. (B) Effects of the sequence between the 534th and 556th amino acid and the sequence between the 295th and 304th on enzyme activity. The TCEP oxidase activity of the variants was relative to that of the wildtype enzyme. The TCEP oxidase activity was based on determining the rate of the H_2_O_2_ generation [22]. The truncated enzyme HsQSOX1b_295–556_ showed high oxidase activity to TCEP, which was slightly higher than that of the full enzyme HsQSOX1b_30–604_. The deletion of the sequence between the 295th and 304th amino acid greatly led to the decrease in the TCEP oxidase activity.

To explore the smallest active fragment of the HsQSOX1b further, we then performed a more detailed truncation of the amino acids between D295-P320 and V524-Q556 (Fig. 2A) and investigated their effects on TCEP oxidase activity (Fig. 2B). The results showed that the deletion of several amino acid residues (HsQSOX1b_295–556_, HsQSOX1b_302–556_, and HsQSOX1b_304–556_) located in the N-terminal alpha-helix of the HRR led to the decrease in protein stability and loss of the FAD binding (Fig. S1, S2), which in turn, resulted in the great decrease of TCEP oxidase activity (Fig. 2B). This finding indicates that the integrity of the HRR domain was indispensable for the structure and the TCEP oxidase activity. The V524-S534 at the C-terminal region, located immediately downstream of the C509–C512 motif, was a crucial region for maintaining the structure and oxidase activity. In particular, the oxidase activity greatly decreased with the deletion of the sequence F^535^SPS^538^ (HsQSOX1b_295–538_, HsQSOX1b_295–536_). The variants with additional deletion of amino acids at the C-terminus, such as HsQSOX1b_295–534_ and HsQSOX1b_295–523_, did not fold well (Fig. S1), could not bind FAD (Fig. S2), and thus lost any oxidase activities (Fig. 2B and Table S2).

Finally, the SAQ, HsQSOX1b_295–540_, was obtained. SAQ showed high TCEP oxidase activity but not the thiol and protein thiol oxidase activities, (Fig. 2B and Table 1). A fragment of HsQSOX1b (HsQSOX1b_286–546_) was recently crystallized and its structure was resolved [16]. However, Alon et al. did not measure the enzymatic activity of this truncated variant. The SAQ, a little bit smaller than HsQSOX1b_286–546_, maintained the TECP oxidase activity, although the thiol oxidase activity was lost. It suggested that HsQSOX1b_286–546_ was active in *de novo* disulfide formation, and this would strengthen the biological significance of its crystal structure. Moreover, the SAQ remained highly active to oxidize the artificial TCEP substrate, suggesting that it is the oxidative engine in HsQSOX1b.

**Table 1:** Comparison of characteristic of QSOX-relative sulfhydryl oxidase.

	QSOX	QSOX267–604	QSOX295–604	SAQ	Erv1c	Erv2c
Dimer	−	−	−	Single-chain pseudo-dimer	Homodimer	Homodimer
Oxidize client protein	+	−	−	−	−	−
Oxidize DTT	+	−	−	−	+	+
Oxidize TCEP	+	+	+	+	+	+
*K_m_* (TCEP, mM)	22.4±2.5	22.9±2.9	21.7±1.5	26.0±4.8	40.9±6.5	2.1±0.1
*k_cat_* (TCEP, TN_max_·min^−1^)	8637±760	5641±501	4765±357	4765±709	147±17	48±2

“+” represents the detected activity;

“−” represents undetected activity;

Turnover numbers (TNs) are quoted in terms of the *de novo* disulfide formation per minute.

### SAQ was Highly Efficient in Catalysis Without Co-operating with Trx Domains

Table 1 compares the catalytic activity of SAQ with HsQSOX1b, Erv1c, and Erv2c using different substrates. Similar to the intact enzyme, SAQ showed much higher oxidase activity toward the non-thiol TCEP substrate, compared with Erv1c or Erv2c. However, without a partner oxidoreductase domain, e.g., the Trx domain, SAQ failed to oxidize either protein thiols or small molecular thiols such as DTT. Our previous studies showed that the ERV/ALR domain active pocket was too extended and too deep to bind the DTT [12]. This result suggests that DTT could not be directly oxidized by the active site of the ERV/ALR domain. In addition, the distal disulfide is located on the opposite side of the protein, 27 Å through the protein core, and a greater distance circumferentially from the active site, as shown by the HsQSOX1b_286–546_ structure [10]. Consequently, we propose that SAQ failed to oxidize DTT because of its inability to directly bind to the active site of the ERV/ALR domain and lacking the assistance of the electron shuttling between the C449–C512 and C509–512 motifs.

Subsequently, we determined the *k_m_* and *k_cat_* values of these enzymes for TCEP (Table 1). The *k_cat_* value of SAQ was about 100-higher than that of the Erv2c, whereas that was 50% of that of the full-length enzyme. In addition, the *k_cat_* values of the variants HsQSOX1b_267–604_ and HsQSOX1b_295–604_ were higher than that of SAQ and much higher than that of the Erv1 or Erv2. These results suggest that SAQ, acted as the oxidative engine, show high efficiency in the *de novo* disulfide formation and oxygen reduction, including the reduction of the C449–C452 disulfide and the electron transfer from this active site to FAD and then to oxygen. Very recently, the higher activity of QSOXs was said to be due to the synergy between the Trx and ERV/ALR domains for disulfide formation [17]. However, this synergy between Trx and ERV domains is only one step for QSOXs to oxidize the thiol and proteineous thiol substrates and only one of the reasons to become a highly efficient enzyme toward protein substrate. Here we demonstrate that another step in the catalysis, which is the *de novo* disulfide formation and oxygen reduction, could also contribute to the high efficiency of QSOXs. We therefore propose that the high efficiency of QSOXs in the oxidation of reduced protein substrate is due to two reasons, one is the SAQ highly efficient “oxidative engine” for *de novo* disulfide formation, and the other is the synergy between domains that transfer the *de novo* formed disulfide to the substrate.

**Figure 3:**
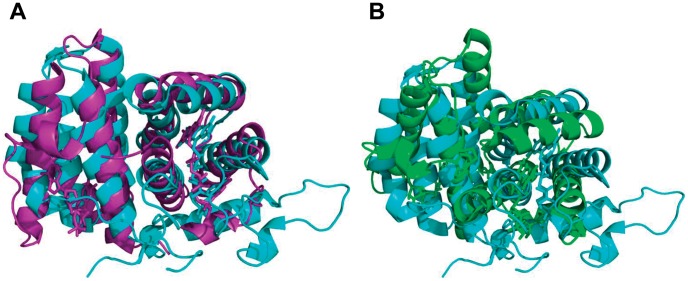
Structural alignment of HsQSOX_286–546_ and the Erv family of sulfhydryl oxidases using PyMol. (A) Structural overlays of HsQSOX_286–546_ and Erv1c. The pale yellow cartoon represents the structure of the HsQSOX_286–546_, and the pale yellow stick is its prosthetic group. The purple cartoon represents the structure of Erv1c, and the purple stick is its prosthetic group. (B) Structural overlays of HsQSOX_286–546_ and Erv2c. The green cartoon represent the structure of Erv2c, and the green stick is its prosthetic group.

**Figure 4:**
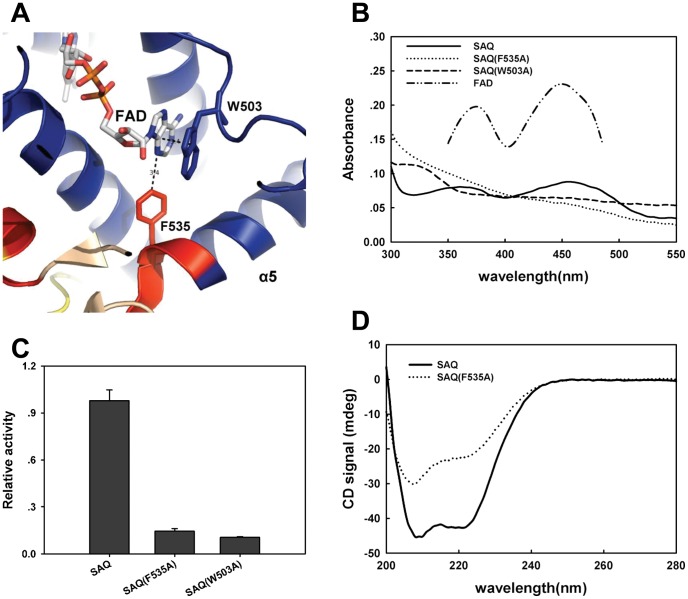
Dissecting the structure and function of the α5 helix of SAQ. (A) The potential sites that bind the FAD purine ring were analyzed using PyMol. (B) UV/visible spectra of SAQ, SAQ F535A, SAQ W503A and FAD. The concentration of SAQ and its variants was 0.3 mg/mL, whereas that of FAD was 5 µM. An absorbance maximum for SAQ was observed in the range of 450 nm, but not for the two mutants. (C) Relative activity of SAQ, SAQ F535A, and SAQ W503A. The mutant variants were only 10% to 20% of the SAQ activity. (D) CD spectra of SAQ and SAQ F535A. The CD signal, as a function of wavelength, is displayed for SAQ and SAQ F535A at the same concentration.

The Structure alignment of HsQSOX1b_286–546_ with the Erv1c or Erv2c homodimer [18], [19] using PyMol showed that the root mean square (RMS) value of the overlay of HsQSOX1b_286–546_ and Erv1c (2.901) was much lower than that of the overlay of HsQSOX1b_286–546_ and Erv2c (12.076) (Fig. 3), suggesting that SAQ was more homologous to Erv1c than to Erv2c. However, we found that the ERV/ALR domain contained a longer α5 helix in SAQ, whereas the HRR domain lacked the relevant helix. We also had confirmed that the intact α5 helix was necessary for maintaining the structure and catalytic activity of HsQSOX1b. To evaluate the function of the α5 helix, we analyzed the crystal structure of HsQSOX1b_286–546_ using PyMol. The distance between purine ring of FAD and benzene ring of the F535 or W503 residue was 3.4 Å (the canonical 3.6 Å) (Fig. 3A). The deletion of F^535^SPS^538^ was assumed to have weakened the binding of FAD and decreased the activity by losing F535. To address the role of F535 and W503, we prepared two SAQ mutants, namely, SAQ F535A and SAQ W503A. The results show that both mutants weakened the binding to FAD (Fig. 4B) and decreased the oxidase activity (Fig. 4C). We further determined the effect of the F535A mutation of SAQ on protein conformation by measuring the circular dichroism (CD) spectroscopy. The ratio of the alpha helix decreased from ∼1.0 to ∼0.83, as calculated according to a previous study using K2d software (Fig. 4D) [20]. Thus, F535 was a critical site for maintaining the active center structure and binding of FAD.

### Conclusions

QSOXs are the only currently known thiol oxidase capable of oxidizing client protein substrates efficiently and directly. In this study, we investigated the structure and function of HsQSOX1b through truncation and established the smallest active truncated fragment of its enzymatic activity. Our results showed that the *de novo* disulfide bond formation of proteins and small molecular thiols depended on the Trx1 domain, whereas the oxidative engine formed by the HRR and ERV/ALR domain, retained the TCEP oxidase activity. Our data also showed that approximately 50 amino acids of the C-terminal tail participated in the regulation of the activity, whereas the ERV/ALR domain α5 helix participated in the FAD-binding pocket formation and was crucial for the folding of the oxidative engine. These results suggested that the crystal structure of a partial QSOX1 [16] was biologically significant for the elucidation of the structure and function of QSOXs.

SAQ showed high oxidase activity to the non-thiol substrate and a hundred-fold higher *k_cat_* than that of Erv1c or Erv2c. These results suggest that SAQ, acted as the oxidative engine, show high efficiency in the *de novo* disulfide formation and oxygen reduction, including the reduction of the C449–C452 disulfide and the electron transfer from this active site to FAD and then to oxygen. Therefore, the oxidative engine, SAQ, is necessary for the highly efficient catalysis of QSOXs. Consequently, we propose that the high efficiency of QSOXs in the oxidation of reduced protein substrate is due to two reasons, one is the SAQ highly efficient “oxidative engine” for *de novo* disulfide formation, and the other is the synergy between domains. Almost all QSOXs orthologs contained both HRR and ERV/ALR domains, which are conserved domains. Thus, we speculated a common mechanism of QSOXs in which the HRR and ERV/ALR domains formed the efficient oxidative engine and then co-operated with the Trx domains to insert the disulfide bonds into the substrate. These findings clearly revealed the function of the different regions in QSOXs, and encouraged the engineering of novel sulfhydryl oxidases, which show efficient catalysis and are more applicable in protein folding.

## Methods

### Vector Construction and Protein Expression

The desired HsQSOX1b_30–604_ cDNA, which omits the signal sequence, was amplified through PCR and inserted into a pETsumo expression vector (Invitrogen). The desired cDNAs of the C-terminal fragment of 15KD ScErv1 (Erv1c) and the ScErv2-core (Erv2c) were inserted into a pGEX-4T-1, both of which contained GST-tag (Table S1). All constructs were identified correctly via restriction enzyme digestion and sequencing.

Using pETsumo HsQSOX1b_30–604_ as template, we obtained the truncated HsQSOX1bs and its mutant variants according to the instructions of the manufacturers’ protocols of the TaKaRa MutanBEST kit (Text S1). All constructs were fully sequenced to verify and ensure that no additional changes in the sequence had been introduced. All truncated proteins were named through their amino acid boundaries (Table S1).

### Expression and Purification of Enzymes

The expression and purification of enzymes was described in Text S2. The recombinant HsQSOX1b, Erv1c and Erv2c constructs were expressed in the *E.coli* strain *Rossetta* (DE3) (Novagen). The HsQSOX1b and its variants were purified using the HisTrap column and HiTrap SP columns (both from GE Healthcare). Erv1c and Erv2c enzymes were purified by Glutathione Sepharose 4B (from GE Healthcare) and an ÅKTA Purifier. The purity of the target proteins was determined through 10% SDS-PAGE analysis.

### Activity Determination of HsQSOX1b and its Truncated Variants

The activity assays were performed at 25°C in a 50 mM potassium phosphate buffer and 1 mM EDTA at pH 7.5 using a BioTek Synergy 2 multifunction microplate reader (USA) and following the continuous fluorescence assay method [12], [21]. Briefly, HsQSOX1b and its variants were determined in 500 nM using the final assay mixture containing 1 mM homovanillic acid (HVA), 1.4 µM horseradish peroxidase (HRP), and 5 mM thiol from DTT, 5.7 mM TCEP, or 300 µM thiol from rRNase. In this assay, oxidase activity was followed by fluorescence of HVA dimer, which was generated from the peroxidase-mediated HVA oxidation by H_2_O_2_. H_2_O_2_ is the produced when oxygen was reduced by substrates like DTT, reduced RNase and TECP, which is catalyzed by QSOX and its truncated variants, and the increase of HVA dimer fluorescence was inhibited by the addition of catalase (data no shown). Basing on determining the rate of the generation of H_2_O_2_, kinetic data were obtained by monitoring the fluorescence production, with 360 nm excitation and 485 nm emission, every 30 s for 10 min [22]. The controls were run without an enzyme. The molar concentrations of the enzymes were decided according to the molecular weight determined using the molar extinction coefficient from the ExPASy proteomics server (http://www.expasy.org/) (Table S2). The preparation of reduced RNase was conducted based a previous study [23]. To determine *k_m_* and *k_cat_*, we formulated the HsQSOX1b and its variant in 100 nM, while Erv1c and Erv2c were formulated in 1 µM. The following the concentrations of TCEP were used: 1.425, 2.85, 4.275, 5.7, 8.55 and 11.4 mM. The other conditions were established according to the abovementioned fluorescence assay method.

### UV/Vis Spectroscopy and CD

The absorption spectra were obtained using a BioTek Synergy 2 multifunction microplate reader blanked with an assay buffer of 50 mM potassium phosphate at pH 7.5 and 1 mM EDTA at 25°C. CD was performed on a Chirascan CD Spectrometer (Applied Photophysics). The samples were diluted to 0.9 mg/mL using 25 mM Tris and 100 mM NaCl at pH 7.5. The spectra were recorded in a 1 mm pathlength cuvette at 25°C.

## Supporting Information

Figure S1 **The yields of the variants after two-step purification from the cells harvested from 100 ml LB.** The yields of the HsQSOX1b_267–604_, HsQSOX1b_295–604_, HsQSOX1b_30–573_, HsQSOX1b_30–556_, HsQSOX1b_295–573_, HsQSOX1b_295–556_ and SAQ were higher than wildtype enzyme; while the yields of the variants without intact HRR or helix α5 in ERV/ALR domain were very low. The yield of HsQSOX1b_295–536_ with low activity was higher than non-activity variants but greatly lower than SAQ.
(TIF)

Figure S2 **UV/visible spectra of the truncated HsQSOX1b variants.** The spectra were recorded in 50 mM potassium phosphate buffer, containing 0.3 mM EDTA, pH 7.5.
(TIF)

Figure S3 **The effect of the C509–C512 motif on the oxidase activity toward three types of substrates.** The oxidase activity of HsQSOX_30–556_ and its C509S–C512S mutant relative to wildtype enzyme. The mutation of C509–C512 motif did not affect the TCEP oxidase activity, whereas the thiol and proteineous thiol oxidase activities were decreased over 60%.
(TIF)

Table S1 **The truncated HsQSOX1bs designed and the primers used in the experiment.**
(DOC)

Table S2 **The activity of all truncated variants was determined.**
(DOC)

Text S1 **Vectors construction.**
(DOC)

Text S2 **Expression and purification of HsQSOX1b truncated variants.**
(DOC)

Text S3 **Expression and activity of HsQSOX1b truncated variants.**
(DOC)
