# Supplementary figures and images for: Exploring the Smallest Active Fragment of HsQSOX1b and Finding a Highly Efficient Oxidative Engine
Source: PLoS One. 2012 Jul 20;7(7):e40935. doi: 10.1371/journal.pone.0040935 (PMC3401233; doi:10.1371/journal.pone.0040935)

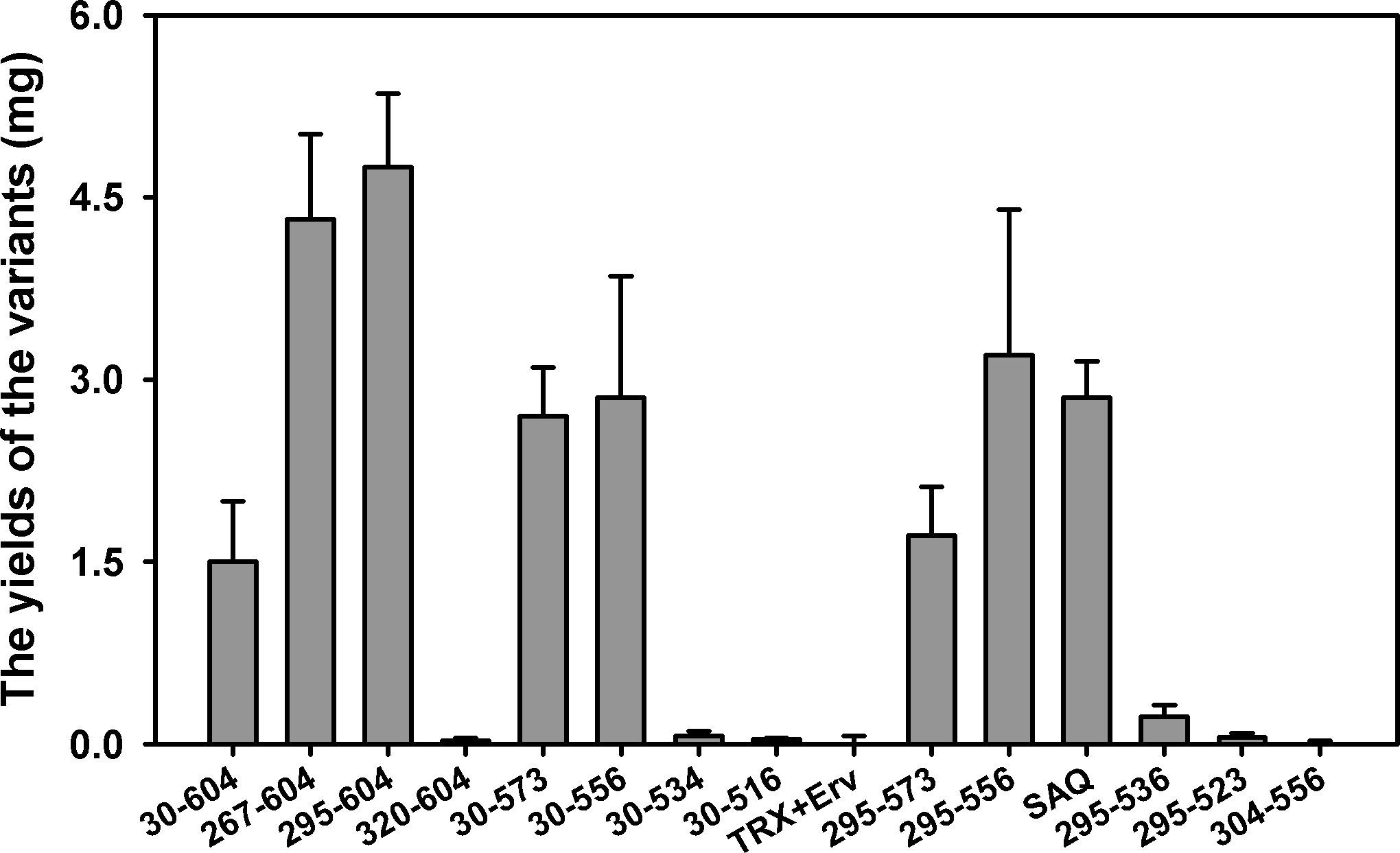

Supplement: Figure S1 — The yields of the variants after two-step purification from the cells harvested from 100 ml LB. The yields of the HsQSOX1b267–604, HsQSOX1b295–604, HsQSOX1b30–573, HsQSOX1b30–556, HsQSOX1b295–573, HsQSOX1b295–556 and SAQ were higher than wildtype enzyme; while the yields of the variants without intact HRR or helix α5 in ERV/ALR domain were very low. The yield of HsQSOX1b295–536 with low activity was higher than non-activity variants but greatly lower than SAQ. (TIF) [file pone.0040935.s001.tif]

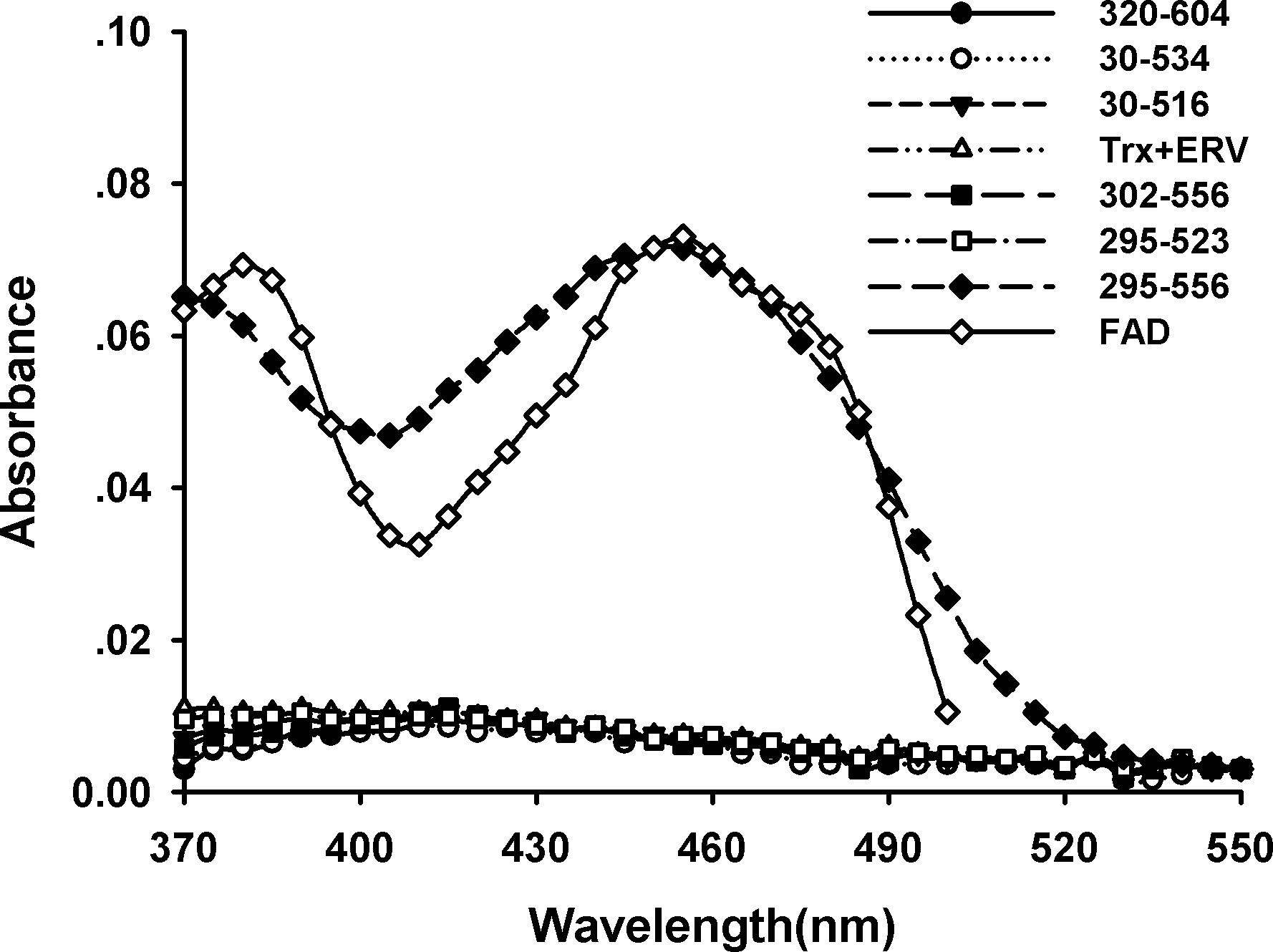

Supplement: Figure S2 — UV/visible spectra of the truncated HsQSOX1b variants. The spectra were recorded in 50 mM potassium phosphate buffer, containing 0.3 mM EDTA, pH 7.5. (TIF) [file pone.0040935.s002.tif]

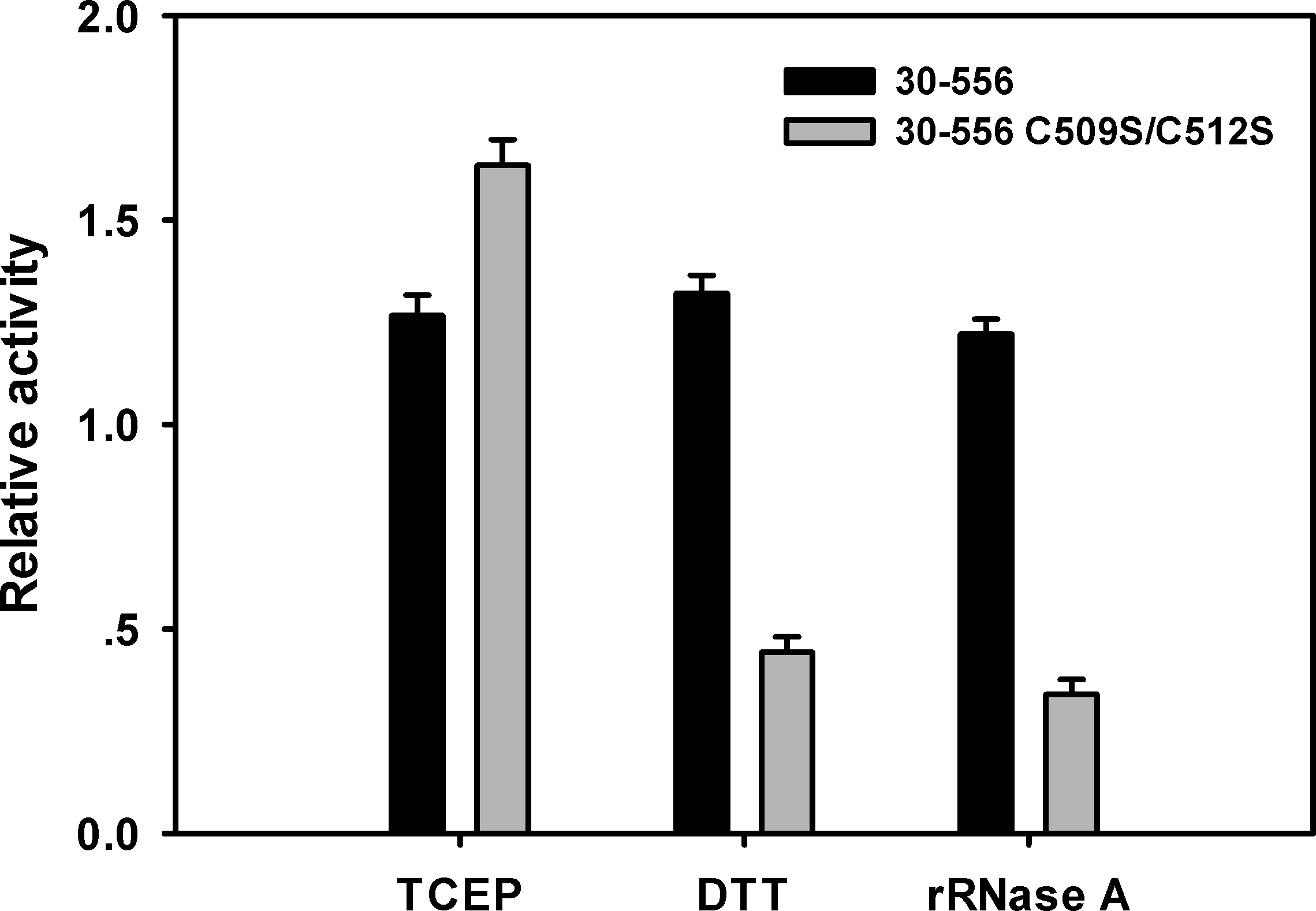

Supplement: Figure S3 — The effect of the C509–C512 motif on the oxidase activity toward three types of substrates. The oxidase activity of HsQSOX30–556 and its C509S–C512S mutant relative to wildtype enzyme. The mutation of C509–C512 motif did not affect the TCEP oxidase activity, whereas the thiol and proteineous thiol oxidase activities were decreased over 60%. (TIF) [file pone.0040935.s003.tif]
