# Supplementary material for: Exploring the Smallest Active Fragment of HsQSOX1b and Finding a Highly Efficient Oxidative Engine
Source: PLoS One. 2012 Jul 20;7(7):e40935. doi: 10.1371/journal.pone.0040935 (PMC3401233; doi:10.1371/journal.pone.0040935)
Supplement: Table S1 — The truncated HsQSOX1bs designed and the primers used in the experiment. (DOC) [file pone.0040935.s004.doc]

**Table S1.** The truncated HsQSOX1bs designed and the primers used in the experiment

| **Name of trunt** | **Amino acid boundaries and character** | **Length (AA)** | **primer** |
| --- | --- | --- | --- |
| HsQSOX1b_30-604_ | 30-604, no signal peptide | 575 | Forward: 5’-atggccccgcggtcggcgctctattcgcctt-3’  Reverse: 5’-tcaaataagctcaggtccctcag-3’ |
| HsQSOX1b_187-604_ | 187-604, no signal peptide and Trx1 domain | 418 | p187N: 5’-ctggctctgatctttgaaaagggaggc-3’  pSUMO1: 5’-accaccaatctgttctctgtgagcctc-3’ |
| HsQSOX1b_210-604_ | 210-604 | 395 | p210: 5’-cacaaaggcgtggcggtgcgcagggtgctga-3’  pSUMO1: 5’-accaccaatctgttctctgtgagcctc-3’ |
| HsQSOX1b_233-604_ | 233-604 | 372 | p233: 5’-gacttcccctcttgctacctgctgttcc-3’  pSUMO1: 5’-accaccaatctgttctctgtgagcctc-3’ |
| HsQSOX1b_267-604_ | 267-604,no signal peptide, Trx1 and Trx2 domain | 338 | p267: 5’-tctgggctcaccagggaggctgcccag-3’  pSUMO1: 5’-accaccaatctg ttctctgtgagcctc-3’ |
| HsQSOX1b_295-604_ | 295-604 | 310 | p295: 5’- gatcgctccaagatctacatggctgacctg-3’  pSUMO1: 5’-accaccaatctgttctctgtgagcctc-3’ |
| HsQSOX1b_320-604_ | 320-604 | 285 | p320: 5’-ccggtcctggaagggcagcgcctggtg-3’  pSUMO1: 5’-accaccaatctgttctctgtgagcctc-3’ |
| HsQSOX1b_343-604_ | 343-604 | 262 | p343: 5’-ggccggcccttagtccagaacttcctg-3’  pSUMO1: 5’-accaccaatctgttctctgtgagcctc-3’ |
| HsQSOX1b_360-604_ | 360-604 | 245 | p360: 5’-aggcagaagagaaataaaattccctacag-3’  pSUMO1: 5’-accaccaatctgttctctgtgagcctc-3’ |
| HsQSOX1b_381-604_ | 381-604, no signal peptide, Trx1, Trx2 and HRR region | 224 | p381: 5’-ggtgccgttcttgccaagaaggtgaactgg-3’  pSUMO1: 5’-accaccaatctgttctctgtgagcctc-3’ |
| HsQSOX1b integrant | 30-186 and 381-604, no signal peptide and HRR region |  | p186down: 5’-tcctgctcctgctcctgcctcttcgttatttctcgcaaag-3’  p381: 5’-gcaggagcaggagcaggaggtgccgttcttgccaagaagggtgaa-3’ |
| HsQSOX1b_30-490_ | 30-490, C-terminal truncated | 461 | p2-490: 5’-tatgcggccgctcaacctgcaaggcgagcattgaccctgttgt-3’  pSUMO2: 5’-agacaagcttaggtatttattcggcgcaaagtgc-3’ |
| HsQSOX1b_30-516_ | 30-516, C-terminal truncated | 487 | p2-516: 5’-tcagcgttcattgtggcaggcagaacaaag-3’  pSUMO2: 5’-agacaagcttaggtatttattcggcgcaaagtgc-3’ |
| HsQSOX1b_30-534_ | 30-534, C-terminal truncated | 505 | p2-534: 5’-tcagtgggccttgaggaagttgagggtggcttc-3’ pSUMO2: 5’-agacaagcttaggtatttattcggcgcaaagtgc-3' |
| HsQSOX1b_30-556_ | 30-556, C-terminal truncated | 527 | p2-556: 5’-tcactgcacatccctccgggcagctgac-3’  pSUMO2: 5’-agacaagcttaggtatttattcggcgcaaagtgc-3’ |
| HsQSOX1b_30-573_ | 30-573, C-terminal truncated | 543 | p2-573: 5’-tcagctttccagctccagggc-3’  pSUMO2: 5’-agacaagcttaggtatttattcggcgcaaagtgc-3’ |
| HsQSOX1b_295-556_ | 295-556, N-, C-terminal truncated | 262 | p295: 5’-gatcgctccaagatctacatggctgacctg-3’  p2-556: 5’-tcactgcacatccctccgggcagctgac-3’ |
| HsQSOX1b_302-556_ | 302-556, N-, C-terminal truncated | 255 | p302: 5’-ctggaatctgcactgcactacatcc -3’  p2-556: 5’-tcactgcacatccctccgggcagctgac-3’ |
| HsQSOX1b_304-556_ | 304-556, N-, C-terminal truncated | 253 | p304: 5’-tctgcactgcactacatcc-3’  p2-556: 5’-tcactgcacatccctccgggcagctgac-3’ |
| HsQSOX1b_311-556_ | 311-556, N-, C-terminal truncated | 246 | p311: 5’-ctgcggatagaagtgggcaggttc-3’  p2-556: 5’-tcactgcacatccctccgggcagctgac-3’ |
| HsQSOX1b_295-546_ | 295-546, N-, C-terminal truncated | 252 | p295: 5’-gatcgctccaagatctacatggctgacctg-3’  p2-546: 5’-tcatgcagggaagtccaggatgatg-3’ |
| HsQSOX1b_295-544_ | 295-544, N-, C-terminal truncated | 250 | p295: 5’-gatcgctccaagatctacatggctgacctg-3’  p2-544: 5’-ctagaagtccaggatgatgttgcttg-3’ |
| HsQSOX1b_295-542_ | 295-542, N-, C-terminal truncated | 248 | p295: 5’-gatcgctccaagatctacatggctgacctg-3’  p2-542: 5’-ctacaggatgatgttgcttggggag-3’ |
| HsQSOX1b_295-542_(SAQ) | 295-540, N-, C-terminal truncated | 246 | p295: 5’-gatcgctccaagatctacatggctgacctg-3’  p2-540: 5’-ctagatgttgcttggggagaagtgg-3’ |
| HsQSOX1b_295-538_ | 295-538, N-, C-terminal truncated | 244 | p295: 5’-gatcgctccaagatctacatggctgacctg-3’  p2-538: 5’-ctagcttggggagaagtgggccttg-3’ |
| HsQSOX1b_295-536_ | 295-536, N-, C-terminal truncated | 242 | p295: 5’-gatcgctccaagatctacatggctgacctg-3’  p2-536: 5’-ctaggagaagtgggccttgaggaag-3’ |
| HsQSOX1b_295-523_ | 295-523, N-, C-terminal truncated | 229 | p295: 5’-gatcgctccaagatctacatggctgacctg-3’  p2-523: 5’-ctagtcccacacgggcacatccag-3’ |
| SAQ F535A | SAQ mutant | 246 | Forward: 5’-gcctccccaagcaacatctag-3’  Reverse: 5’-gtgggccttgaggaagttgag-3’ |
| SAQ W503A  HsQSOX1b_30-556_ C509S C512S | SAQ mutant  C509-C512 mutant | 246 | Forward: 5’-gcgccaccccgtgaactttg-3’  Reverse: 5’-ctgcaccttggggaactg-3’  Forward: 5’-gccagccacaatgaacgcctggatg-3’  Reverse: 5’-agaactaagttcacggggtggccactg-3’ |

Note: The truncated variants were named according to their amino acid boundaries.
