# Supplementary material for: Exploring the Smallest Active Fragment of HsQSOX1b and Finding a Highly Efficient Oxidative Engine
Source: PLoS One. 2012 Jul 20;7(7):e40935. doi: 10.1371/journal.pone.0040935 (PMC3401233; doi:10.1371/journal.pone.0040935)
Supplement: Table S2 — The activity of all truncated variants was determined. (DOC) [file pone.0040935.s005.doc]

**Table S2.** The activity of all truncated variants was determined.

| **Name of truncated proteins** | **Molar extinction coefﬁcient(ɛ)** | **Oxidase activity (TCEP)** | **Thiol-oxidase activity (DTT)** | **Protein-thiol oxidase activity(rRNase)** | **Yield** | **FAD binding** |
| --- | --- | --- | --- | --- | --- | --- |
| HsQSOX1b30-604 | 91245 | **+** | **+** | **+** | High | Y |
| HsQSOX1b187-604 | 60578 | **+** | **-** | **-** | High | Y |
| HsQSOX1b267-604 | 53128 | **+** | **-** | **-** | High | Y |
| HsQSOX1b295-604 | 57598 | **+** | **-** | **-** | High | Y |
| HsQSOX1b30-556 | 91245 | **+** | **+** | **+** | High | Y |
| HsQSOX1b30-573 | 91245 | **+** | **+** | **+** | High | Y |
| HsQSOX1b295-556 | 47628 | **+** | **-** | **-** | High | Y |
| HsQSOX1b295-546 | 47628 | **+** | **-** | **-** | High | Y |
| HsQSOX1b295-544 | 47628 | **+** | **-** | **-** | High | Y |
| HsQSOX1b295-542 | 47628 | **+** | **-** | **-** | High | Y |
| HsQSOX1b295-540(SAQ) | 47628 | **+** | **-** | **-** | High | Y |
| HsQSOX1b295-538 | 47628 | **+** | **-** | **-** | High | Y |
| HsQSOX1b295-536 | 47628 | + | **-** | **-** | High | Y |
| HsQSOX1b integrant | 66773 | + | + | ND | Low | N |
| HsQSOX1b30-534 | 91245 | + | + | + | Low | N |
| HsQSOX1b320-604 | 44648 | **-** | **-** | **-** | Low | N |
| HsQSOX1b343-604 | 43158 | **-** | **-** | **-** | Low | N |
| HsQSOX1b360-604 | 37658 | **-** | **-** | **-** | Low | N |
| HsQSOX1b381-604 | 36168 | **-** | **-** | **-** | Low | N |
| HsQSOX1b30-490 | 80183 | **-** | **-** | **-** | Low | N |
| HsQSOX1b30-516 | 85745 | **-** | **-** | **-** | Low | N |
| HsQSOX1b295-523 | 47628 | **-** | **-** | **-** | Low | N |
| HsQSOX1b302-556 | 46138 | **-** | **-** | **-** | Low | N |
| HsQSOX1b304-556 | 46138 | **-** | **-** | **-** | Low | N |

Note:

“+” represents detected activity ;

“-“ represents undetected activity;

“+” represents very low activity;

“ND” represents not detected;

“Y” represents binding FAD;

“N” represents lacking FAD.
