# Supplementary material for: Exploring the Smallest Active Fragment of HsQSOX1b and Finding a Highly Efficient Oxidative Engine
Source: PLoS One. 2012 Jul 20;7(7):e40935. doi: 10.1371/journal.pone.0040935 (PMC3401233; doi:10.1371/journal.pone.0040935)
Supplement: Text S1 — Vectors construction. (DOC) [file pone.0040935.s006.doc]

**Vectors construction**

The desired HsQSOX1b_30-604_ cDNA, which omits the signal sequence, was amplified through PCR with a forward primer and a reverse primer incorporating a stop codon. The PCR products were directly ligated into a pETsumo expression vector (Invitrogen) containing a SUMO tag in the N-terminal of target protein and identified correctly by restriction enzymes digestion and sequencing.

Using pETsumo HsQSOX1b30-604 as template, we obtained the truncated HsQSOX1bs according to the instructions of the manufacturers’protocols of the TaKaRa MutanBEST kit. The PCR fragments containing the desired sequence were phosphorylated and directly ligated into the circle vector and transformed into E.coli DH5α. The mutants of SAQ, W503A and F535A were prepared by site-directed mutations with the following primer pairs: W503A forward and reverse primers; F535A forward and reverse primers. All constructs were sequenced to verify and ensure that no additional changes in the sequence had been introduced.
