# Supplementary material for: Exploring the Smallest Active Fragment of HsQSOX1b and Finding a Highly Efficient Oxidative Engine
Source: PLoS One. 2012 Jul 20;7(7):e40935. doi: 10.1371/journal.pone.0040935 (PMC3401233; doi:10.1371/journal.pone.0040935)
Supplement: Text S2 — Expression and purification of HsQSOX1b truncated variants. (DOC) [file pone.0040935.s007.doc]

**Expression and purification of HsQSOX1b truncated variants**

The recombinant HsQSOX1b constructs were expressed in the *E.coli* strain Rossetta (DE3) (Novagen). Cells were grown in LB media containing 100 mg/L kanamycin and 34 mg/L chloramphenicol to an OD600 of 0.6–0.8 at 37℃. Isopropyl β-D-1-thiogalactopyranoside (IPTG) and flavin adenine dinucleotide (FAD) were added to a final concentration of 0.3 mM and 10 μM, and the cultures were grown for a further 20 h at 18℃. Cells were harvested at 4000 rpm for 20 mins and resuspended in the Buffer A (50mM sodium phosphate buffer containing 500 mM NaCl, and 10 mM imidazole, pH 7.4), and sonicated on ice, then centrifuged for 20 min at 10000 rpm at 4℃. The supernatant was applied to a HisTrap column using an ÅKTA FPLC (both from GE Healthcare), and protein was eluted in Buffer A and a gradient of imidazole (20-500 mM) (pH 7.4). The eluted protein fractions were collected and incubated with 10 μM FAD for 24h at 4℃. The pooled protein fractions were loaded onto the HiTrap SP columns and eluted with 0-1 M NaCl gradient. Active fractions were pooled and ultrafiltrated against 100 mM sodium phosphate buffer, containing 300 mM NaCl, pH 7.0. The purity of target proteins was determined through 10% SDS-PAGE analysis.
