# Supplementary material for: Exploring the Smallest Active Fragment of HsQSOX1b and Finding a Highly Efficient Oxidative Engine
Source: PLoS One. 2012 Jul 20;7(7):e40935. doi: 10.1371/journal.pone.0040935 (PMC3401233; doi:10.1371/journal.pone.0040935)
Supplement: Text S3 — Expression and activity of HsQSOX1b truncated variants. (DOC) [file pone.0040935.s008.doc]

**Expression and activity of HsQSOX1b truncated variants**

*E.coli* Rossetta (DE3) (Novagen) was employed to produce truncated HsQSOX1b variants. High yields of active soluble enzymes were obtained as the induction temperature was lowered to 18℃ from the growth temperature 37℃, following by the resuspension of the cells in fresh media containing 0.3 mM isopropyl-β-D-thiogalactopyranoside (IPTG) and 10 μM FAD, since induction at 37℃ led to expression of the protein as insoluble aggregates. In addition, considering the low level expression and low stability, we constructed the DNA sequence of HsQSOX1b variants in pETsumo vector (invitrogen) in which the variants containing the SUMO fusion tag increasing the solubility.

The truncated HsQSOX1b variants were purified through two step purification processes from the crude lysate followed by HisTrap chromatography and SP-ion exchange chromatography. Typically, the purity of the truncated HsQSOX1bs and fusion proteins, were confirmed by SDS-PAGE with >90% purity. The activity of all truncated HsQSOX1b were determined, showed in Supporting information Table 2.
